# Bloom of the cyanobacterium *Moorea bouillonii* on the gorgonian coral *Annella reticulata* in Japan

**DOI:** 10.1038/srep06032

**Published:** 2014-08-12

**Authors:** Hideyuki Yamashiro, Naoko Isomura, Kazuhiko Sakai

**Affiliations:** 1 Sesoko Station, Tropical Biosphere Research Center, 3422 Sesoko, Motobu, Okinawa 905-0227, Japan; 2 Department of Bioresources, Okinawa National College of Technology, Henoko 905, Okinawa 905-2192, Japan

## Abstract

Coral populations are in decline due to environmental changes and biological attacks by predators and infectious diseases. Here, we report a localized bloom of the benthic filamentous cyanobacterium *Moorea bouillonii* (formerly *Lyngbya bouillonii*) observed exclusively on the gorgonian (sea fan) coral *Annella reticulata* at around 20 m depth in Japan. The degree of infection has reached 26% among different sizes of *Annella* colonies. Thick and continuous growth of *Moorea* may be sustained partly by symbiotic alpheid shrimp, which affix *Moorea* filaments to gorgonian corals for use as food and shelter. Most filaments get entangled on the coral colony, some penetrate into the stem of the coral with a swollen end like a root hair, which appears to function as an anchor in *Annella*. In addition to the cyanobacterium–shrimp interaction, the new trait of anchoring by the cyanobacterium into gorgonian coral may contribute to persistence of this bloom.

Populations of reef-building coral have been in decline due to biological and physiological changes, such as predation by crown-of-thorns starfish and other species, coral bleaching driven by increasing water temperature, a variety of diseases, and ocean acidification^1^,^2^,^3^,^4^,^5^. It has been predicted that, in regions with coral reefs, a phase shift from coral to algae will occur due to climate change and anthropogenic activities^1^,^3^. Algal (including cyanobacteria) blooms, which are usually caused by excess nutrient input into reefs from terrestrial systems^4^, suppress both coral growth and coral recruitment^5^. Algae–coral interactions have been of increasing interest because algae can produce poisonous chemicals and vector coral diseases^6^,^7^,^8^.

Harmful cyanobacterial blooms have received increasing attention because they pose a serious threat to the use and sustainability of freshwater and marine resources^9^,^10^. Most cyanobacterial blooms in coral reefs are caused by inputs of nutrients; examples include guano containing phosphorus at the Great Barrier Reef, Australia^11^, and nitrogen inputs into watersheds of Florida, USA^4^,^12^,^13^. In addition to nutrient-induced cyanobacterial blooms, some cyanobacteria cause coral diseases. Black band disease (BBD) is a well-known coral disease, caused by cyanobacteria and a complex consortium of other bacteria^14^. Soft corals (octocorals), as well as hard corals, have experienced higher rates of infectious diseases in recent years. The fungus *Aspergillus sydowii* causes the disease aspergillosis and exclusively kills the common Caribbean octocoral *Gorgonia ventalina*^15^,^16^,^17^.

*Moorea bouillonii* is a common benthic filamentous cyanobacterium distributed widely such as Papua New Guinea, Guam, Palau, Palmyra atoll etc.^18^, French Polynesia^19^ and Japan^20^. We report on a localized cyanobacterial bloom in a colony of sea fan coral, *Annella* *reticulata* (Anthozoa, Alcyonacea, Gorgoniidae), caused by a common benthic cyanobacterium, *M. bouillonii*, which proliferates in the water near Okinawa, Japan.

## Results

**Figure 1:**
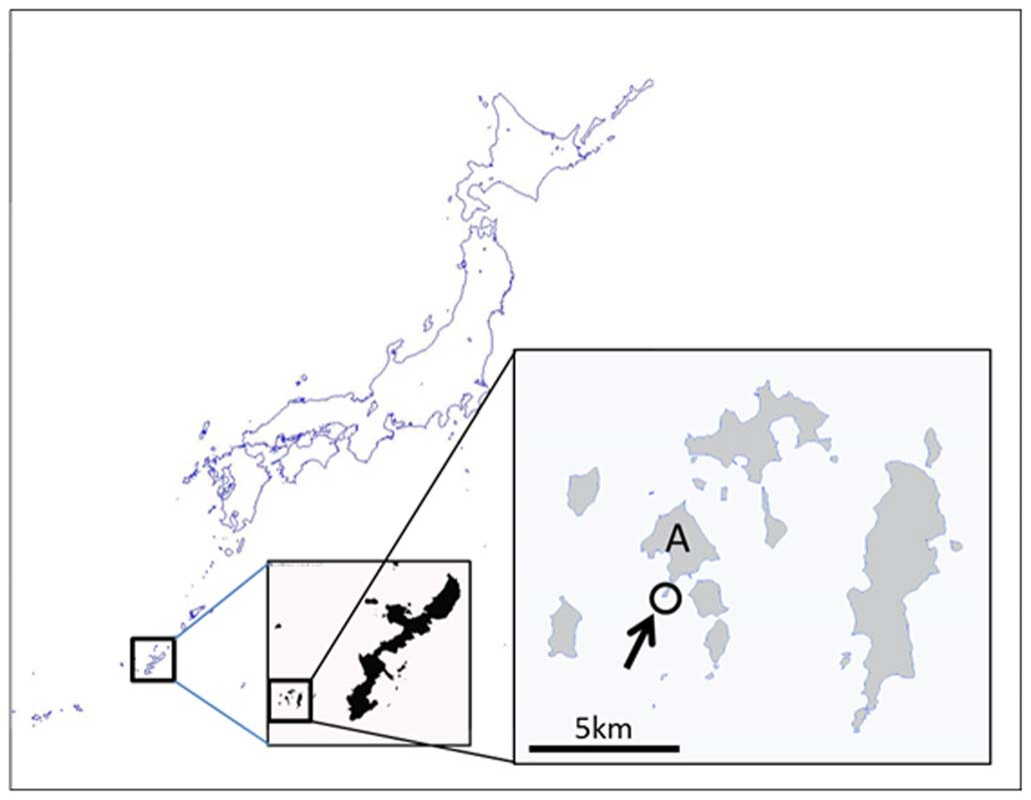
Map showing the study site. Bloom of the cyanobacterium *Moorea bouillonii* on the gorgonian coral *Annella reticulata* was observed at Sakubaru reef (arrow) in Akajima Island (A). Maps were downloaded from The Geospatial Information Authority of Japan.

**Figure 2:**
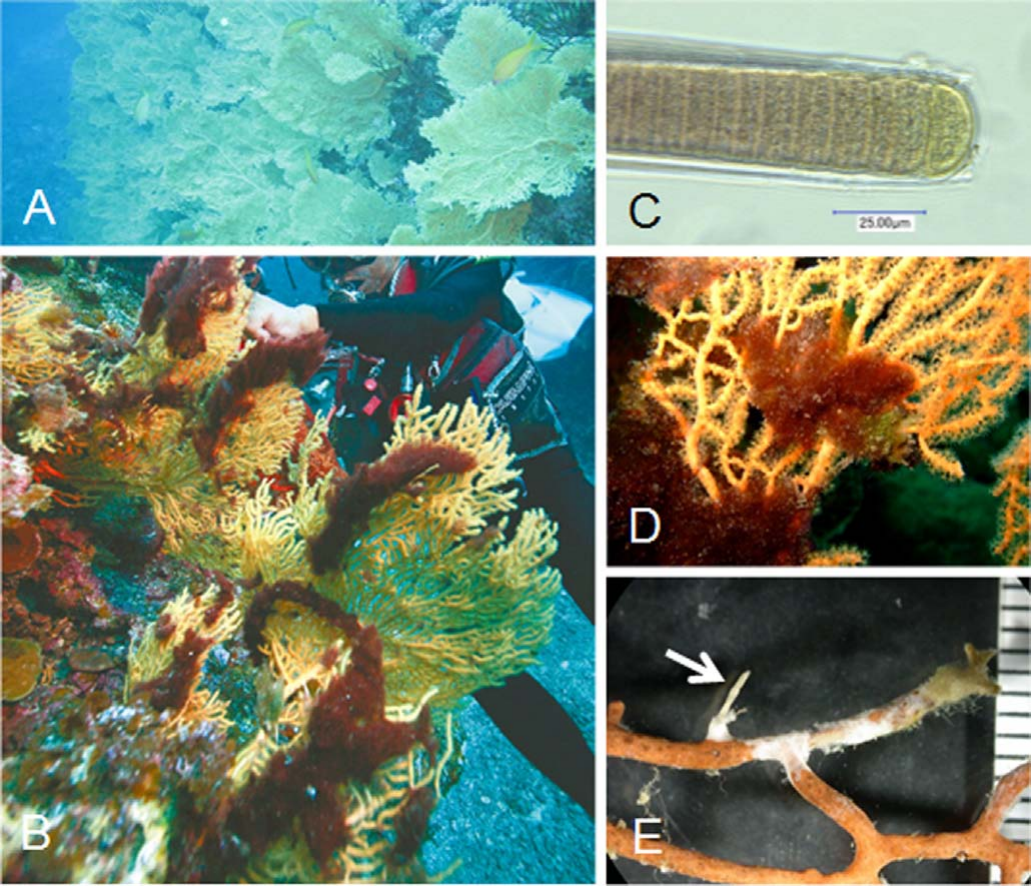
(A), The colonies of gorgonian coral *Annella reticulata*. (B), Overgrown by the cyanobacterium *Moorea bouillonii.* (C), Enlarged view of *M. bouillonii*. (D), Close-up of the coral branches entangled by *Moorea*. (E), After removal of *M. bouillonii* filaments, dead branch (white) part and collapsed branch leaving only a central axis (arrow) are shown.

This study was conducted at Sakubaru-reef, Aka-jima, Okinawa, Japan (26°10′37″ N, 127°16′ 27″E), which is approximately 1.5 km from the residential area of Aka-jima (Fig. 1). This is a deep (>20 m), clear-water environment where gorgonian corals (sea fans) are dominant and scleractinian corals also occur along the reef slope. The gorgonian coral *Annella* *reticulata* (Fig. 2A) forms a dense population approximately 50 m wide by 100 m long, at 10–25 m depth (A. Kishi, pers. com.). Attachment of filamentous cyanobacteria to gorgonian coral was first observed about 10 years ago at 20 m depth and has gradually increased in both number and in extent since then (H. Matayoshi, personal observation). Cyanobacterial overgrowth was observed exclusively on the colony surface of the gorgonian coral and was not found on other substratum or sessile organisms including scleractinian corals (Fig. 2B,D).

The cyanobacterial alga was identified as *Moorea bouillonii* (Basionym: *Lyngbya bouillonii* Hoffmann & Demoulin, 1991)^18^,^22^,^23^ by its color, cell size (Fig. 2C), and 16S ribosomal RNA sequence. Partial 16S ribosomal RNA sequence of it (about 680 bp, Accession No. AB922817) supported that the cyanobacteria belonged to the genus *Moorea*. The gorgonian coral was identified by its colony structure and sclerites as *Annella reticulata* (Ellis and Solandar, 1786).

**Figure 3:**
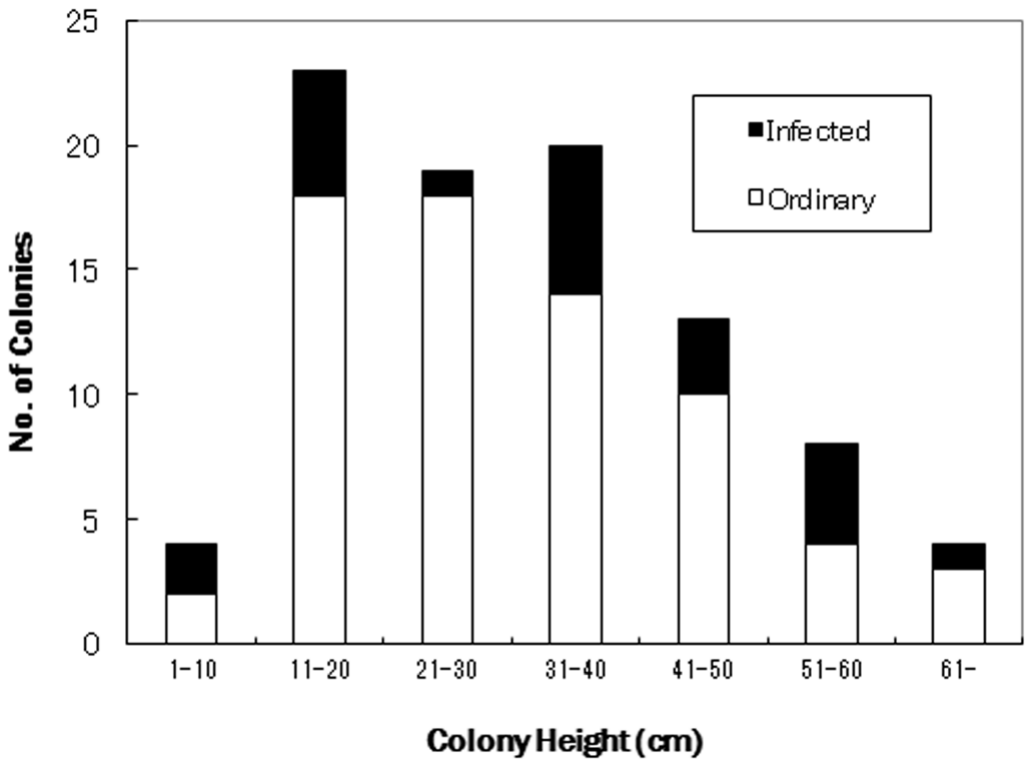
Percentage of infection of the cyanobacterium *Moorea bouillonii* on the gorgonian coral *Annella reticulata* at a depth of 18 ± 1 m (n = 91).

Percentage of infected colonies of *M*. *bouillonii* on *A*. *reticulata* reached 26%, and some algal cover was found on every size class of coral colony (Fig. 3). A small amount of other algal species (filamentous green or attached diatoms) was observed, but *M*.*bouillonii* was the most abundant and entangled on branches of *Annella*. The sewing (tube-forming) shrimp, *Alpheus frontalis* H. Milne Edwards 1958, was identified by the presence of tubular cyanobacteria, but quantitative measurement was not performed because most shrimp escaped from the cyanobacterial tubes during collection. Other small organisms found in the cyanobacterial mat, which were considered to be secondarily attached, included foraminiferas, nematodes, copepods, gastropods, and tunicates etc. Nutrient concentrations measured from the sea surface to 25 m in depth were 0.17 µmol for NH_4_, 0.04 µmol for NO_2_, 0.99 µmol for NO_3_, and 0.09 µmol for PO_4_.

**Figure 4:**
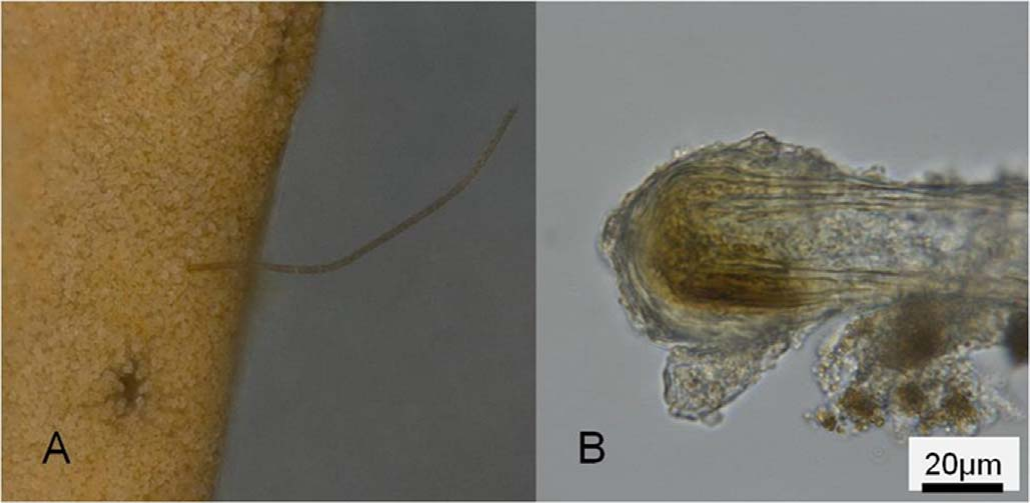
(A), Penetration by the cyanobacterium *Moorea bouillonii* into host coral *Annella reticulata*. (B), Terminal end of *M. bouillonii* showing swallen structure composed of multi-layer of sheath.

Coral branches overgrown by *Moorea* mats ultimately die, which results in collapse of the branch (Fig. 2E) followed by gradual detachment of outer sclerites and then the loss of successively longer sclerites that form the inner axis. Most *Moorea bouillonii* filaments were entangled on coral colonies, although some were loosely attached and lying on the branch surface. Some filaments penetrated directly into the coral branch and reached the outermost region of the central axis (Fig. 4A). The terminal end of the boring filament was swollen like a hair root, and consisted of a multilayered sheath (Fig. 4B) which functions as an anchor.

## Discussion

Seaweeds negatively impact corals via multiple mechanisms such as shading, abrasion, vectoring of coral diseases, and release of metabolites^8^,^24^. Cyanobacterial blooms in coral reefs are due to excessive anthropogenic nutrient loading, and have been reported from Florida, USA^4^,^13^, Guam^25^, and Queensland, Australia^11^,^26^. The ambient nutrient concentrations measured at this study site (0.99 µmol for NO_3_ and 0.09 µmol for PO_4_) were slightly below the levels previously reported for sustaining macroalgal blooms (1.0 µmol for NO_3_ and 0.1 µmol for PO_4_^27^ or cyanobacterial growth in enrichment experiments^26^,^28^,^29^. On the other hand, it is widely accepted that reefs are not limited to low-nutrient areas^1^,^27^,^30^. Thus, to accurately address algal growth, we must consider nutrients, producers (algae), and consumers (herbivores, predators).

Herbivores consume algae, affecting algae–coral interactions. In a marine protected area in Fiji, the amount of macroalgae is better controlled than in an adjacent fished reef^7^. In general, however, filamentous cyanobacteria and gorgonian corals are generally not preferred food for predators such as benthivorous fish^31^,^32^,^33^. Furthermore, *L*. *majuscula* produces feeding deterrents, such as ypaoamide^34^ and lyngbyatoxin^35^. Cytotoxic macrolides and peptides have been identified from samples of *Moorea bouillonii* associated with *Alpheus frontalis* shrimp in Guam^36^. Similarly, gorgonian corals are not suitable food because they possess chemical metabolites and mechanical sclerites as defenses against fish^37^,^38^, as well as antifungal secondary compounds^39^. Furthermore, among cnidarian animals, sea fan corals develop cell-based immune defenses (amoebocytes)^40^. Nevertheless, gorgonian corals, as well as hard corals, are facing a crisis of fungal infestations (e.g., aspergillosis disease in the Caribbean Sea^17^,^41^) and algal blooms due to eutrophication.

At our study site, how the cyanobacteria initially settled on the coral is unknown, but the important question is how the bloom is maintained in oligotrophic water. Engene et al.^18^ showed that *Moorea* *bouillonii* lacks heterocysts and genes for nitrogen fixation. Nutrient concentrations at the study site were not high enough from the water surface to 25 m deep to cause algal blooms. Furthermore, cyanobacterial coverage was observed exclusively on *Annella*. The tube-forming or sewing shrimp *Alpheus* *frontalis* H. Milne Edwards have been found in cyanobacterial tubes which they made to live and to eat^20^,^21^. Thus, the sewing shrimp *Alpheus* may play an important role in perpetuating continuous blooms by attaching cyanobacterial filaments to coral branches to form tube-like mats that it uses for food and shelter. NH_4_ and PO_4_ excreted from the shrimp were absorbed by *Moorea* determined in a laboratory experiment (not shown, Yamashiro unpublished data). In addition, this shrimp, like other alpheid shrimp, uses its large claws to snap at other animals and protect its nest made of *Moorea*^42^,^43^. This symbiosis also seems to have a synergistic relationship in respect to nutrition (between photosynthetic cyanobacteria and nitrogen/phosphorus-emitting shrimp).

Concerning the mortality of *Annella* due to *Moorea* cover, there are several possible mechanisms. Coral death can be caused by metabolic decline including oxygen depletion or a reduction of food supply to the corals by algal (including cyanobacteria) coverage^44^,^45^,^46^,^47^. In addition to physical stress, biochemical effects such as allelopathic terpenes secreted by algae have been reported as causing coral death^48^. Titlyanov^49^ performed direct contact experiment using cyanobacteria *Lyngbya* (*Moorea*) *bouillonii* on live coral *Porites*, and demonstrated that *M. bouillonii* acted as a one-sided inhibitor for scleractinian corals inducing bleaching and severe damage of live coral tissue. Similar interaction is often observed in the field of Okinawa Island between *M. bouillonii* and branching corals such as *Montipora* nested by sewing shrimp. Coral tissue where filamentous *M. bouillonii* was tied by the shrimp showed bleached and partial death (not shown). The main cause of octocoral death was not identified in this study, physical effects such as abrasion or oxygen depletion, or biochemical (toxic or allelopathic) effects must be involved.

Some cyanobacteria associated with the coral are able to penetrate into the soft tissue and skeleton^50^. Our study highlights that *Moorea bouillonii* is capable of penetrating tissues of gorgonian coral branches by changing its shape at the terminal end. A swollen structure of multiple layers of sheath appears to function as an anchor (Fig. 4B), firmly attaching the cyanobacterium to *Annella*. Strong persistence of *M*. *bouillonii* to the host coral should exist, but this trait has previously been unrecognized. The origin and transmission of the cyanobacterium is still unknown, but the synergy between filamentous *Moorea* and sewing shrimp, and the special trait of penetration found in this *M*. *bouillonii*, must allow the persistence of year-round blooms on the sea fan *Annella reticulata*.

## Methods

We first collected filamentous algae entangled on the gorgonian coral colony on 3 March 2009. We measured the concentrations of nutrients (NH_4_, NO_3_, NO_2_, and PO_4_) using a nutrient autoanalyzer (BL Tech Co.) of triplicate seawater samples collected every 5 m down to 25 m depth on 10 April 2009. We identified the filamentous algae by morphology and molecular information (16S rRNA sequence, see below), and the gorgonian coral by morphological observation of colony structure and sclerites on live or formalin-fixed samples using dissecting or digital microscopes (VHX-1000, Keyence Co.). We also made histological sections to determine the method of attachment of the filamentous algae.

On 17 September 2009, we recorded the height of all gorgonian corals within a 2-m-wide × 20-m-long transect at 18 m depth on a nearly vertical reef with the highest local density of cyanobacteria infection. We recorded the overgrowth (infection) by filamentous cyanobacteria on the coral on all colonies (n = 91) within the transect and classified them into size classes.

The cyanobacterial alga was washed 2–3 times with filtered seawater and stored in CHAOS solution (4 M Guanidine thiocyanate, 0.5% Salkosyl,25 mM Tris-HCl pH8.0,0.1 M 2-Mercaptoethanol after removing extra seawater(partly modified^51^). Following standard phenol chloroform methods, genomic DNA from the cyanobacterial alga was extracted.

Cyanobacteria-specific PCR primers CYA106F (CGGACGGGTGAGTAACGCGTGA) and CYA781R (an equimolar mixture of CYA 781R(a) (GACTACTGGGGTATCTAATCCCATT) and CYA781R(b) (GACTACAGGGGTATCTAATCCCTTT)^52^ were used to amplify an about 680-bp region of the 16S rRNA gene. Reaction mixture of 25 µl contained 0.6 µM of each primer, 0.2 mM of each dNTP, 1X PCR Reaction Buffer (TaKaRa), 1.5 mM of MgCl_2_ solution, 0.08% (w/v) bovine serum albumin, 0.2 U of ExTaq DNA Polymerase (TaKaRa) and 20 ng of template DNA. Amplification was performed with initial melting at 94°C for 3 min, followed by 30 cycles of 94°C for 1.5 min, 59°C for 1 min and 72°C for 2 min, and a final extension at 72°C for 5 min. After electrophoresis, PCR products were purified with DNA Cleaner (Wako). The purified PCR products were cloned using TOPO cloning kit (Invitrogen). The totals of twenty clones of sequences were carried out on an automated sequencer CEQ8800 (Beckman Coulter).

## Author Contributions

H.Y. designed the entire study, performed most of the experiments, and wrote the paper. N.I. performed genetic analysis. K.S. conducted field observation of infected octocorals.
